# Blood parasite load by qPCR as therapeutic monitoring in visceral leishmaniasis patients in Brazil: a case series study

**DOI:** 10.1590/0037-8682-0456-2022

**Published:** 2023-03-27

**Authors:** Samuel Ricarte de Aquino, Lucyo Flávio Bezerra Diniz, Sávio Luiz Pereira Nunes, Roberta Lane de Oliveira Silva, Gisele Veneroni Gouveia, João José Simoni Gouveia, Kamila Gaudêncio da Silva Sales, Filipe Dantas-Torres, Rodrigo Feliciano do Carmo

**Affiliations:** 1 Universidade Federal do Vale do São Francisco, Programa de Pós-Graduação em Ciências da Saúde e Biológicas, Petrolina, PE, Brasil.; 2 Universidade Federal do Vale do São Francisco, Hospital Universitário, Petrolina, PE, Brasil.; 3 Universidade de Pernambuco, Programa de Pós-Graduação em Biologia Celular e Molecular Aplicada, Recife, PE, Brasil.; 4 Universidade Federal do Vale do São Francisco, Laboratório de Microbiologia Geral, Petrolina, PE, Brasil.; 5 Universidade Federal do Vale do São Francisco, Colegiado de Zootecnia, Petrolina, PE, Brasil.; 6 Instituto Aggeu Magalhães, Departamento de Imunologia, Recife, PE, Brasil.

**Keywords:** Kala-azar, Leishmania infantum, qPCR, Relapse, Visceral leishmaniasis

## Abstract

**Background:**

This study aimed to describe the kinetics of *Leishmania* parasite load determined using kinetoplast DNA (kDNA)-based quantitative polymerase chain reaction (qPCR) in visceral leishmaniasis (VL) patients.

**Methods:**

Parasite load in blood was assessed by qPCR at five time points, up to 12 months post-diagnosis. Sixteen patients were followed up.

**Results:**

A significant reduction in the parasite load was observed after treatment (*P* < 0.0001). One patient had an increased parasite load 3 months post-treatment and relapsed clinically at month six.

**Conclusions:**

We have described the use of kDNA-based qPCR in the post-treatment follow-up of VL cases.

Visceral leishmaniasis (VL) is a chronic, neglected disease caused by intracellular protozoa belonging to the genus *Leishmania*. In the Indian subcontinent, Asia, and Africa, VL is caused by *Leishmania donovani* and is an anthroponotic disease (i.e., interhuman transmission)^1^. In Latin America, VL is a zoonosis caused by *Leishmania infantum*, which is mainly transmitted through the bites of female phlebotomine sand flies of the species *Lutzomyia longipalpis* ^2^.

Relapse is a new VL episode that occurs after an initial cure, usually within 12 months of the initial treatment^3^. Currently, relapse monitoring is performed by assessing clinical signs and symptoms after the administration of the last dose of drug therapy. Patients who relapse need to undergo a new spleen, bone marrow, or lymph node biopsy procedure to confirm the presence of parasites. These are invasive, costly, and potentially hazardous techniques with variable sensitivities, ranging from 53 to 99%^4^.

One of the main challenges in the treatment of VL is the monitoring and prediction of relapse. Quantitative polymerase chain reaction (qPCR) using peripheral blood samples is an important tool for monitoring treatment response^5^ ^-^ ^9^. Previous studies adopted variable follow-up periods, and meglumine antimoniate was the most commonly used drug. In the present study, we used qPCR to quantify *Leishmania* kinetoplast DNA (kDNA) levels in peripheral blood at the time of diagnosis and during follow-up to evaluate this method for monitoring treatment relapses.

The study was conducted in a high-complexity hospital located in the city of Petrolina, Pernambuco, Brazil (University Hospital of the Universidade Federal do Vale do São Francisco). The patients were followed-up during the infectious disease service of the municipality.

Petrolina is located in the São Francisco Valley, in the Northeast Region of Brazil. It is considered a hyperendemic region for VL, with a mean incidence rate of 4.4 human cases per 100,000 inhabitants between 2007 and 2017^10^, in addition to very high *Leishmania* positivity in dogs^11^.

The study population consisted of individuals aged ≥ 15 years (due to the hospital's profile) who sought health care for a clinical and laboratory condition suspected of VL between July 2017 and July 2020. The cases were confirmed by parasite visualization in bone marrow aspirates. Patients were included in the analysis if they had their blood samples collected before treatment and had at least one follow-up visit after treatment. All patients were tested for human immunodeficiency virus (HIV). 

During follow-up, patients were clinically evaluated at baseline (T0), one day after treatment completion (T1), 3 months (T2), 6 months (T3), and 12 months (T4). Blood samples were collected at each visit for the quantification of *Leishmania* kDNA using qPCR. In addition, bone marrow samples were collected at baseline for parasite quantification.

This study was approved by the Ethics Committee of the Universidade Federal do Vale do São Francisco (CAAE:68562617.3.0000.5196).

After diagnosis, all patients started VL therapy following the recommendations of the Brazilian Ministry of Health. In Brazil, the drugs used for the treatment of VL are N-methyl glucamine antimoniate and amphotericin B. For the choice of medication, age group, pregnancy, and comorbidities are considered. N-methyl glucamine antimoniate was administered at a dose of 20 mg/Sb+5/kg/day, intravenously or intramuscularly, once a day, for 30 days. Liposomal amphotericin B (L-AmB) was intravenously administered at a dose of 4 mg/kg/day for 5 days. Patients with HIV-LV co-infection received secondary prophylaxis with L-AmB at a dose of 4 mg/kg every 15 days. Clinical response was defined as remission of fever, improvement in hematological values, and regression in spleen and/or liver size during therapy. Clinical relapse was defined as the recurrence of VL symptoms and signs within 12 months of treatment.

Samples were collected in EDTA tubes (Vacuette K3EDTA tube, Greiner Bio-One, Kremsmünster, Austria) and DNA extraction was performed using the PureLink Genomic DNA Mini Kit (Invitrogen, Carlsbad, California, USA) according to the manufacturer’s instructions. The DNA samples were eluted to a final volume of 100 μL. The overall quality of DNA in terms of purity was assessed using a NanoDrop OneC spectrophotometer (Thermo Scientific, Waltham, Massachusetts, USA). DNA samples were frozen at −20 °C until analysis.

DNA samples were screened for the presence of *Leishmania spp*. kDNA by qPCR using the primers LEISH-1 (5’-AACTTTTCTGGTCCTCCGG GTAG-3’) and LEISH-2 (5’-ACCCCCAGTTTCCCGCC-3’), as well as the TaqMan-MGB probe (FAM-5’-AAAAATGGGTGCAGAAAT-3’-nonfluorescent quencher-MGB), as described previously^12^. The reaction mixture contained 7.5 μL of GoTaq® Probe qPCR Master Mix (2×) (Promega, Madison, Wisconsin, USA), 1.35 μL of each primer (final concentration of 900 nM each), 0.3 μL of probe (final concentration of 200 nM each), 2.5 μL of sterile water (DNase and RNase free), and 2.0 μL of genomic DNA (< 100 ng per reaction), in a final volume of 15 μL. The qPCR thermal conditions were as follows: 50°C for 2 min, 95°C for 10 min, and 40 cycles of 95°C for 15 s, and 60°C for 1 min. All assays were performed using the QuantStudio 5 real-time PCR system (Applied Biosystems, Foster City, California, USA).

A standard curve prepared using nine serial dilutions (1 ng, 100 pg, 10 pg, 1 pg, 100 fg, 10 fg, 1 fg, 0.1 fg, 0.01 fg per reaction) of genomic DNA extracted from cultured *L. infantum* promastigotes was used as a positive control and also to estimate the parasite load in the patient samples. DNA-free water was used as a non-template control. All qPCR reactions were performed in duplicates. 

Statistical analyses and graph preparation were performed using GraphPad Prism version 8 (GraphPad Software, Inc., San Diego, California, USA). The Kolmogorov-Smirnov test was used to investigate the normality of the data distribution. Statistical differences in median parasite load were determined using the Wilcoxon signed-rank test. Statistical significance was set at *P* < 0.05.

During the study period, 38 patients were diagnosed with VL. Four (10.5%) patients died before the end of treatment (median of 1.46 log parasites/mL at T0, data not shown). Sixteen patients fulfilled the inclusion criteria and were followed-up before (T0) and after treatment (T1). Nine patients were lost to follow-up at T2 (3 months), one at T3 (6 months), and only one who presented with clinical symptoms at T3 returned at T4 (12 months). 

The baseline clinical and laboratory characteristics of the patients are presented in Supplementary Table 1. The age of the patients ranged from 19 to 67 years (mean = 43.1); men were more prevalent (81.3%), and three patients (18.7%) had VL-HIV coinfection (Supplementary Table 1). All VL-HIV patients were receiving antiretroviral therapy at the time of VL diagnosis.

*Leishmania* kDNA was detected in all 16 patients with VL at the time of diagnosis (T0), with a median of 2.56 log parasites/mL (range 0.71-4.09). The parasite load in the bone marrow of eight patients with available samples was found to be approximately two times higher than that observed in blood samples at T0 (*P* = 0.007) (Supplementary Figure 1), corroborating previous findings^13^. 

Thirteen patients were treated with L-AmB (81.2%) and three with N-methyl glucamine antimoniate (Table 1). 

**TABLE 1:** Clinical characteristics and parasite load determined by qPCR in blood samples at baseline (T0), after treatment (T1), 3 months (T2), 6 months (T3), and 12 months (T4).

					***Leishmania* kDNA (log parasites/mL)**				
Sample ID	Sex	Age (Years)	HIV/VL Coinfection	Drug	T0	T1	T2	T3	T4
1	M	42	No	MA	1.91	0.00	0.00	0.00	-
2	M	57	No	L-AmB	2.66	0.30	-	-	-
3	F	39	No	L-AmB	4.01	1.14	-	-	-
4	M	33	No	L-AmB	1.57	0.14	-	-	-
5	F	57	No	L-AmB	0.71	0.11	0.00	0.00	-
6	M	51	No	L-AmB	2.75	0.35	0.00	0.00	-
7	M	19	No	L-AmB	2.54	0.20	-	-	-
8	M	42	No	MA	1.23	0.07	-	-	-
9	M	27	No	L-AmB	2.15	1.48	0.00	0.21	-
10	M	49	No	L-AmB	3.42	0.54	0.00	0.17	-
11	M	67	No	L-AmB	1.06	0.02	2.28	4.11	1.90
12	M	26	No	L-AmB	2.59	0.27	-	-	-
13	M	43	No	MA	1.67	0.00	-	-	-
14	F	52	Yes	L-AmB	4.09	3.14	-	-	-
15	M	34	Yes	L-AmB	2.89	1.69	-	-	-
16	M	53	Yes	L-AmB	2.78	1.03	0.00	-	-

**F:** female; **L-Amb:** liposomal amphotericin B; **M:** male; **MA:** meglumine antimoniate.

A significant reduction in parasite load was observed at T1 compared to T0 (median, 2.56 vs. 0.28 log parasites/mL; Wilcoxon signed-rank test, *P* < 0.0001) (Figure 1). Two patients (12.5%) presented with undetectable parasitemia at T1. Despite the small number of VL-HIV coinfected patients, it was possible to observe that these individuals showed a persistently high parasite load at T1 (median, T0 = 2.89, T1 = 1.69 log parasites/mL; *P* = 0.25) (Figure 1).

**FIGURE 1:**
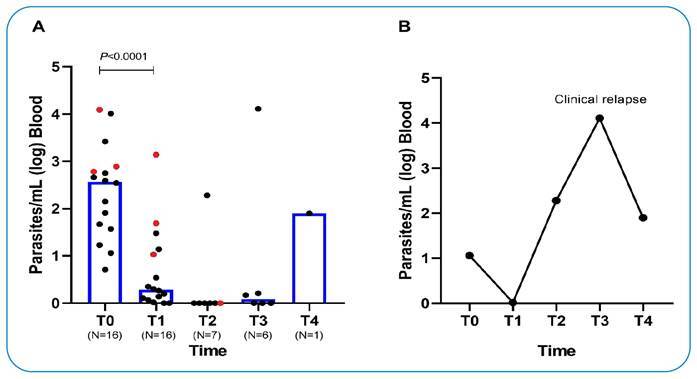
Parasite load determined by qPCR in blood samples at the baseline (T0), after treatment (T1), at 3 months (T2), 6 months (T3), and 12 months (T4). **(A)** Bars represent the median parasite load (log parasites/mL). **(B)** Follow-up of a patient who presented relapse after treatment. Statistical significance was determined using Wilcoxon signed-rank test. Circles in red represent individuals with VL-HIV coinfection.

At T2 and T3, the median parasite load was 0.00 and 0.08 log parasites/mL, respectively. Only patient number 11 had an increased parasite load at T2, but had no noticeable clinical signs or symptoms at the time of the visit. At T3, the patient returned to the clinic presenting with asthenia, weight loss, hepatomegaly, and worsening hematological parameters. The patient was treated again with L-AmB. At T4, after the second treatment, the patient no longer had symptoms and there was a reduction in the parasite load (Figure 1). The clinical and laboratory characteristics of this patient are shown in Supplementary Table 2.

The present study demonstrates that *Leishmania* kDNA detection by qPCR in blood samples may be a potential target in the diagnosis and post-treatment follow-up of patients in areas endemic for VL.

We observed a significant reduction in the parasite load immediately after the end of treatment. Despite this reduction, only two patients (12.5%) had undetectable *Leishmania* kDNA levels at T1. At T2 (3 months), six of seven patients (85.7%) had undetectable *Leishmania* kDNA levels. Previous studies have demonstrated the usefulness of qPCR in estimating the parasite load in blood samples of VL patients and also observed that despite the significant reduction in parasite load at the end of treatment, it remains detectable in most patients^5^ ^-^ ^9^. Meglumine antimoniate is the most commonly used therapeutic regimen, and a significant reduction in parasite load is observed during and after treatment^6^ ^,^ ^7^. In the present study, more than 80% of patients were treated with L-AmB.

Bossolasco et al. (2003)^9^ used qPCR to follow-up the parasite load of ten patients with HIV-VL coinfection treated with L-AmB. They observed a significant drop in parasite load after five days of treatment. Seven patients clinically relapsed a median of 110 days after the end of treatment, in association with substantial increases in *Leishmania* DNA levels. Mary et al. (2004)^5^ observed a significant drop in Leishmania DNA after treatment with Ambisome^®^, which remained at less than 1 parasite/mL. In addition, Aoun et al. (2009)^6^ observed a significant reduction in parasite load after treatment with meglumine antimoniate, where 23 of 39 patients (58.9%) tested negative immediately after treatment completion. Long-term samples (day 180) obtained from nine of these patients showed a continuous decrease, with negative parasite load in seven samples. Finally, Pourabbas et al. (2013)^7^ demonstrated that after completing meglumine antimoniate treatment, parasites were cleared from the peripheral blood in the majority (76%) of patients. Two weeks after the discontinuation of treatment, it was cleared in almost all patients (95%); finally, on day 90, it was cleared in all patients. 

Despite the methodological differences between the studies (e.g., timing of collection, parasite genetic target, and PCR methods), the treatment regimen may partly explain the differences in clearance time between the studies, as treatment with L-AmB is administered for 5 days, and treatment with meglumine antimoniate can range between 20 and 40 days. In this study, two of three (66.6%) patients treated with meglumine antimoniate had undetectable *Leishmania* kDNA levels immediately after the completion of treatment. 

In our study, a 67-year-old patient showed a 2-log increase in parasite load 3 months after treatment, and relapsed clinically at month six. Advanced age has been associated with relapse in immunocompetent patients treated with L-AmB^3^. In addition, previous studies have observed increased post-treatment parasitemia in individuals who relapsed. Sudarshan et al. (2014)^14^ observed an increase in *L. donovani* parasitemia within 30 days of treatment with L-AmB in individuals who relapsed, corroborating other studies that observed an increase above 10 parasites/mL in blood before clinical relapse^5^ ^,^ ^9^. Recently, Verrest et al. (2021)^8^ demonstrated that the absolute parasite load on day 56 was a highly sensitive predictor of relapse at a cut-off of 20 parasites/mL. These findings support blood parasite load determined by qPCR as a promising biomarker for predicting relapse in VL patients.

This study had some limitations, including the small sample size and significant loss to follow-up. However, few studies have evaluated the role of therapeutic monitoring by qPCR in the blood of individuals infected with *L. infantum* using L-AmB. This study presents data on the detection of *Leishmania* kDNA using qPCR in a series of cases followed-up longitudinally after therapy. One case of clinical relapse presented an increased parasite load before symptom onset. Further studies with larger populations are needed to evaluate the use of this technique, as well as the definition of a cutoff point, in the prediction of relapse in VL patients.

## Figures and Tables

**SUPPLEMENTARY FIGURE 1:**
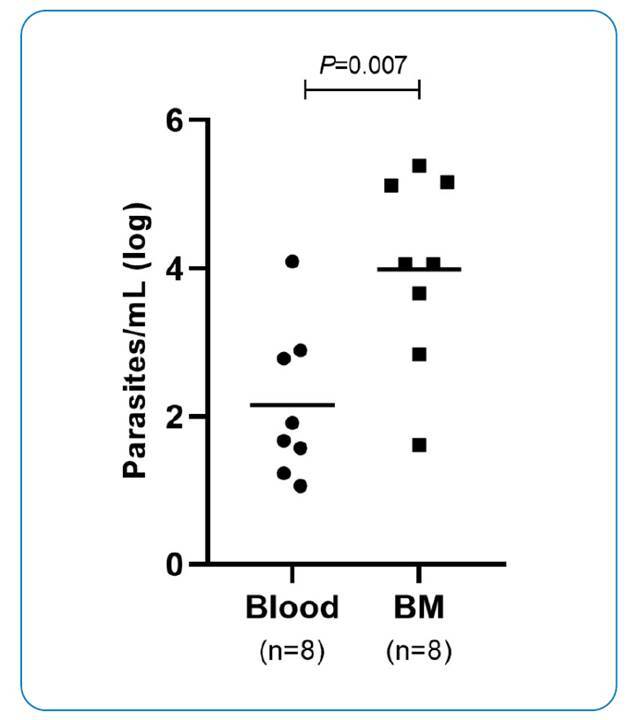
Parasite load determined by qPCR in blood and bone marrow samples. Horizontal line represents the mean parasite load (log parasites/mL). Statistical significance was determined using student’s t test. Mean: Blood = 2.1 parasites/mL, BM: 3.9 parasites/mL. BM, Bone marrow.

## Appendix

**SUPPLEMENTARY TABLE 1:** Baseline characteristics of hospitalized individuals with visceral leishmaniasis included in the study.

Variable	N = 16
Age, years (mean ± SD)	43.1 ± 13.0
***Sex***	
Male	13 (81.3%)
Female	3 (18.7%)
***Comorbidities***	
Chronic kidney disease	1 (6.3%)
Chronic heart disease	1 (6.3%)
HIV	3 (18.7%)
***Symptoms***	
Fever	12 (75.0%)
Weakness	12 (75.0%)
Bleeding	5 (31.2%)
Weight loss	13 (81.3%)
Splenomegaly	16 (100.0%)
Hepatomegaly	12 (75.0%)
***Laboratory parameters****	
Hemoglobin (g/dL)	9.2 (8.6-10.1)
Leucocytes (mm^3^)	2,591 (1,895-2,800)
Platelets (mm^3^)	94,000 (73,750-132,000)
Creatinine (mg/dL)	0.7 (0.6-1.0)
Urea (mg/dL)	25.5 (22.2-30.7)
Total Bilirubin (mg/dL)	1.3 (0.6-2.6)
Aspartate transaminase (U/L)	81.0 (40.5-148.0)
Alanine transaminase (U/L)	43.0 (29.7-94.0)
Albumin (g/dL)	2.5 (2.0-3.3)

*Values are shown as median (interquartile range). **HIV:** human immunodeficiency virus.

## Appendix

**SUPPLEMENTARY TABLE 2:** Clinical and laboratory characteristics of a patient (case 11) with VL relapse after L-AmB treatment.

Variable	
Age (years)	67
Gender	Male
Zone of residence	Rural area
***Comorbidities***	
Chronic kidney disease	Yes
Chronic heart disease	Yes
HIV	No
***Symptoms***	
Fever	Yes
Weakness	Yes
Bleeding	No
Weight loss	Yes
Splenomegaly	Yes
Hepatomegaly	No
***Laboratory parameters***	
Hemoglobin (g/dL)	10.0
Leucocytes (mm^3^)	2180
Platelets (mm^3^)	313000
Creatinine (mg/dL)	1.0
Urea (mg/dL)	30.0
Total Bilirubin (mg/dL)	0.3
Aspartate transaminase (U/L)	80.0
Alanine transaminase (U/L)	40.0
Albumin (g/dL)	2.8

**HIV:** human immunodeficiency virus.
